# A prospective multicenter phase II study on the efficacy and safety of dasatinib in the treatment of metastatic gastrointestinal stromal tumors failed by imatinib and sunitinib and analysis of NGS in peripheral blood

**DOI:** 10.1002/cam4.3319

**Published:** 2020-07-17

**Authors:** Ye Zhou, Xinhua Zhang, Xiaojun Wu, Yongjian Zhou, Bo Zhang, Xiufeng Liu, Xin Wu, Yan Li, Lin Shen, Jian Li

**Affiliations:** ^1^ Department of Gastric Surgery Fudan University Shanghai Cancer Center Shanghai China; ^2^ Department of Gastrointestinal Surgery the First Affiliated Hospital Sun Yat‐sen University Guangdong China; ^3^ Department of Colorectal Surgery Sun Yat‐sen University Cancer Center State Key Laboratory of Oncology in South China Collaborative Innovation Center of Cancer Medicine Guangzhou China; ^4^ Department of Gastric Surgery Union Hospital of Fujian Medical University Fuzhou China; ^5^ Gastrointestinal Surgery West China Hospital Sichuan University Sichuan China; ^6^ People's Liberation Army Cancer Center Bayi Hospital Affiliated to Nanjing University of Chinese Medicine Nanjing China; ^7^ General Surgery General Hospital of the People's Liberation Army Beijing China; ^8^ Department of GI Oncology Laboratory of Carcinogenesis and Translational Research of the Ministry of Education Peking University School of Oncology Beijing Cancer Hospital & Institute Beijing China

**Keywords:** dasatinib, gastrointestinal stromal tumor, next‐generation sequencing

## Abstract

**Aim:**

Dasatinib is a small molecule tyrosine kinase inhibitor with multiple targets including kit, PDGFR, and SRC. This prospective study evaluated the efficacy and safety of dasatinib as third‐line treatment for gastrointestinal stromal tumors (GIST).

**Methods:**

The study enrolled adult patients (≥18 years of age) with histologically confirmed unresectable and/or metastatic GIST whose disease progressed despite imatinib and sunitinib therapy. Dasatinib (50 mg twice daily) was given orally for 1 week and escalated to 70 mg twice daily orally. The primary endpoint was to the 3‐month progression‐free survival (PFS) rate. Blood samples were acquired before dasatinib therapy for examination of gene mutations by next‐generation sequencing (NGS).

**Results:**

From May 2016 to June 2018, 58 patients from 9 Chinese medical centers were enrolled in this study. The 3‐month PFS rate was 53.4% and the median overall survival (OS) was 14.0 months. Neither primary nor secondary gene mutations predicted the efficacy of dasatinib. Wild‐type GIST patients had longer PFS (5.5 months). The most common adverse events were anemia, proteinuria, fatigue, neutropenia, and diarrhea. The concordance of *KIT/PDGFRA* mutation was 61.9% between tissue and peripheral blood samples and additional *KIT* mutations were detected in the peripheral blood samples in 28.6% of the patients. Some SNV and CNV such as *ATRX*, *TP53*, *TEKT4*, *STK11*, *SDHC*, and *CDKN2C* related to tumor signaling pathways were detected. Patients with *TP53* mutations and *SDHC* and *TMEM127* gene copy number loss had longer OS.

**Conclusion:**

Dasatinib has modest antitumor activity with tolerable toxicities in patients with metastatic GISTs who have failed imatinib and sunitinib therapy.

## Graphical abstract

This is the first prospective trial to evaluate the efficacy of dasatinib in third line treatment of GIST and reached the expected primary endpoint. The results of NGS ananlysis and liquid biopsy are also very important to clinical practice.

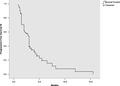

## 1 INTRODUCTION

Gastrointestinal stromal tumor (GIST) is the most common mesenchymal tumor of the digestive tract. Advanced GISTs including unresectable, recurrent or metastatic are initially managed by imatinib; however, GIST patients may have primary resistance to imatinib due to mutations in *KIT* or *PDGFRA* rendering the patients less responsive to imatinib. In addition, resistance to imatinib may emerge due to secondary mutations in GIST patients in whom GISTs progress after an initial response to imatinib.^1^ Although sunitinib and regorafenib as inhibitors of KIT, PDGFRA, and vascular endothelial growth factor receptor kinases have been approved for second‐ and third‐line treatment,^2^, ^3^ their efficacy is not satisfactory, and the number of therapeutic drugs is still insufficient.

Dasatinib is a small molecule, adenosine triphosphate competitive inhibitor of *KIT*, *PDGFR*, and the protooncogene tyrosine‐protein kinase Src (*SRC*) family.^4^ The drug has demonstrated antitumor activities as first‐ and second‐line treatment of GISTs.^5^, ^6^ Trent et al have shown in a phase II study of dasatinib for patients with imatinib‐resistant GIST that 21% patients (10/47) had a progression‐free survival (PFS) >6 months and an overall survival (OS) of 19 months. In a single‐arm clinical trial of 50 patients with advanced GISTs resistant to imatinib, Shuetze et al found that patients with mutations in *KIT* or *PDGFRA* showed varied response to dasatinib. However, the relationship between secondary mutations of *KIT*, the change of downstream pathway status, and the efficacy of dasatinib has not been analyzed and explained in these studies.

Therefore, we carried out this prospective, multi‐center study to evaluate the efficacy and safety of dasatinib in the third‐line treatment of metastatic GISTs. Meanwhile, we performed next‐generation sequencing (NGS) in peripheral blood to analyze gene variations of *KIT* downstream signaling pathway and the mechanism of drug resistance.

## 2 PATIENTS AND METHODS

### 2.1 Patient selection

The study enrolled adult patients (≥18 years of age) with histologically confirmed unresectable and/or metastatic GIST whose disease progressed despite imatinib and sunitinib therapy. The main inclusion criteria were (a) at least one measurable GIST lesion at baseline and (b) an Eastern Cooperative Oncology Group (ECOG) performance status score ≤ 2. We excluded patients who had received tyrosine kinase inhibitors (TKI) within 21 days of initiation of dasatinib therapy. Patients with impaired cardiac function, brain metastasis, or uncontrollable gastrointestinal bleeding were also excluded.

### 2.2 Study design

This open‐label, multi‐center, and single‐arm phase II study evaluated the efficacy of dasatinib for patients with metastatic GIST who had failed imatinib and sunitinib therapy. The primary endpoint was PFS at 3 months of dasatinib therapy. The secondary endpoints included PFS at 6 months, overall response rate (ORR), disease control rate (DCR), OS, and safety. Blood samples before dasatinib therapy were acquired for examination by NGS.

The study protocol adhered to the SPIRIT statement^7^ and all procedures performed in this study involving human participants were approved by Beijing Cancer Hospital Ethics Committee. Written informed consent was obtained from the patients before study entry. The study was conducted according to the Declaration of Helsinki and the reporting of the study adhered to the CONSORT statement.^8^ The clinical trial is registered with Clinicaltrial.gov (NCT02776878).

### 2.3 Study drug administration

Dasatinib (50 mg twice daily) was given orally for 1 week. If tolerable, the dosage of dasatinib was increased to 70 mg twice daily orally until tumor progression, appearance of unacceptable toxicities, or occurrence of death. Each dasatinib treatment cycle lasted 1 month.

### 2.4 Efficacy and safety

Tumor assessment was evaluated by local radiological review according to the Response Evaluation Criteria in Solid Tumors (RECIST v1.1). All the tumor lesions were investigated using computed tomography at baseline within 2 weeks of enrollment before initiating therapy, and then every 6 weeks until disease progression. Physical examination, blood cell counts, blood biochemistry, and urine analysis were carried out at baseline and on day 1 of each cycle. Adverse events (AEs) were recorded according to Common Terminology Criteria for Adverse Events (CTCAE), version 5.0.

### 2.5 Gene mutation examination

All the patients received *KIT/PDGFRA* genotype analysis with GIST tissue before and after imatinib resistance. Genomic DNAs were extracted from paraffin‐embedded tumor specimens using E.Z.N.A. FFPE DNA Kit (Lot. D3399‐1, OMEGA) according to the manufacturer's instructions. DNA fragments were aligned with exons 9, 11, 13, and 17 of *KIT*, and exons 12 and 18 of *PDGFRA*, and amplified by polymerase chain reaction (PCR) using specific primers. Sequencing results were analyzed with Chromas software. For patients with secondary resistance, exons 14 and 18 of *KIT* were also included.

Blood samples before dasatinib therapy were acquired for NGS by GENECAST Technology Co., Ltd.

#### 2.5.1 Library construction and sequencing

Buffy coat and plasma were separated by centrifugation at 1600 *g* for 10 minutes at room temperature. Genomic DNA and cfDNA were extracted from buffy coat and plasma, respectively. Genomic DNA was sheared into 150‐200 bp fragments for DNA libraries construction. DNA libraries were captured with a targeted sequencing panel of 1406 genes that included major tumor‐related genes.

#### 2.5.2 Identification of somatic mutations

VarDict and FreeBayes tools were used for somatic mutation calling with the following filters: (a) location in intergenic regions or intronic regions; (b) synonymous single nucleotide variations (SNVs); (c) allele frequency >0.002 in the database exac03; (d) allele frequency <0.01 in the plasma sample; (e) strand bias mutations in the reads; and (f) support reads <5.

#### 2.5.3 Copy number variation (CNV) calling and copy number instability (CNI) calculation

We used CNV kit (v0.9.2) to call CNV of plasma DNA samples. Paired blood samples were used as the baseline. A copy number ≥2.5 and ≤1.5 (in plasma‐free DNA) was used to categorize altered regions as CNV gains (amplification) and copy number losses (deletions), respectively. After correction for GC content and length of the target region using proprietary algorithms for each region, the read counts were transformed into log2 ratios and converted into Z‐score based on Gaussian transformations vs a normal control group (n = 30). The target regions that satisfied the Z‐score greater than the 95th percentile plus two times absolute standard deviation (SD) of the normal control group were retained, and these Z‐scores were summed as the CNI score.

#### 2.5.4 Quality control

VarDict, a software for detecting PCR artifacts, was used for SNV calling. Strict filter standards were introduced to ensure validation: allele frequency should be less than 0.002 in the database exac and greater than 0.01 in the plasma sample; furthermore, support reads should be greater than 5, and the mutations should be without strand bias.

### 2.6 Statistical considerations

Based on the GRID trial,^3^ the 3‐month PFS rate was 11% in patients who received placebo after imatinib and sunitinib failure. Assuming a 3‐month PFS rate of 50% with dasatinib treatment, a default rate of 10%, and a one‐sided level of significance of 0.05, an overall sample size of 57 subjects was required to achieve 80% power at a 0.05% significance level in a two‐sided log‐rank test.

All statistical analyses were undertaken using SPSS 19.0 (SPSS Inc.). PFS and OS curves were constructed by the Kaplan‐Meier method and compared using a log‐rank test. In order to adjust for confounding variables, Cox proportional hazards models were used to estimate the simultaneous effects of prognostic factors on PFS. Frequency and percentage descriptions were used for categorical variables and chi‐squared test was conducted to compare the incidence of different events. If the theoretical frequency was lower than 1, Fisher's exact test was conducted.

## 3 RESULTS

### 3.1 Patient demographic and baseline characteristics

From May 2016 to June 2018, 58 patients from 9 medical centers across China were enrolled in this study. The median age of the patients at the time of study entry was 55 years (range, 24‐77 years). In all, 43 patients (74.1%) were men, and 35 patients (60.3%) had ECOG performance status score of 0‐1. GIST progressed in all the patients after imatinib and sunitinib treatment failure. Among them, 54 patients completed at least one imaging assessment. All the patients underwent *KIT/PDGFRA* genotyping. In total, 39 patients were examined for secondary gene mutations in GIST tissues from cytoreductive surgery or biopsy of progressing lesion after imatinib treatment failure. In addition, 22 peripheral blood samples before dasatinib treatment were obtained to examine gene mutations in circulating tumor DNA (Table 1).

**TABLE 1:** Clinicopathologic features at baseline

Clinicopathologic features	Number (%)
Gender	
Male	43 (74.1)
Female	15 (25.9)
Ages	55 (24‐77)
Primary location	
Stomach	21 (36.2)
Small intestinal	29 (50.0)
Other	8 (13.8)
ECOG PS	
1	35 (60.3)
2	23 (39.7)
Primary genotype	
Exon 11 mt	36 (62.1)
Exon 9 mt	7 (12.1)
PDGFR D842V	4 (6.9)
Wild type	5 (8.6)
SDHB loss	2 (3.4)
Non‐SDHB loss	3 (5.2)
Other genotype	6 (10.3)
Exon 13	1 (1.7)
Exon 17	2 (3.4)
PDGFR exon 12	1 (1.7)
PDGFR exon 18 D842Y	1 (1.7)
Failed for tissue quality	1 (1.7)
Secondary genotype	
Exon 13/14 mt	5 (8.6)
Exon 17 mt	17 (29.3)
No secondary mutation found	17 (29.3)
Secondary mutation not done	19 (32.8)
NGS in peripheral blood	
Yes	21 (36.2)
No	37 (63.8)

### 3.2 Efficacy measures

**FIGURE 1:**
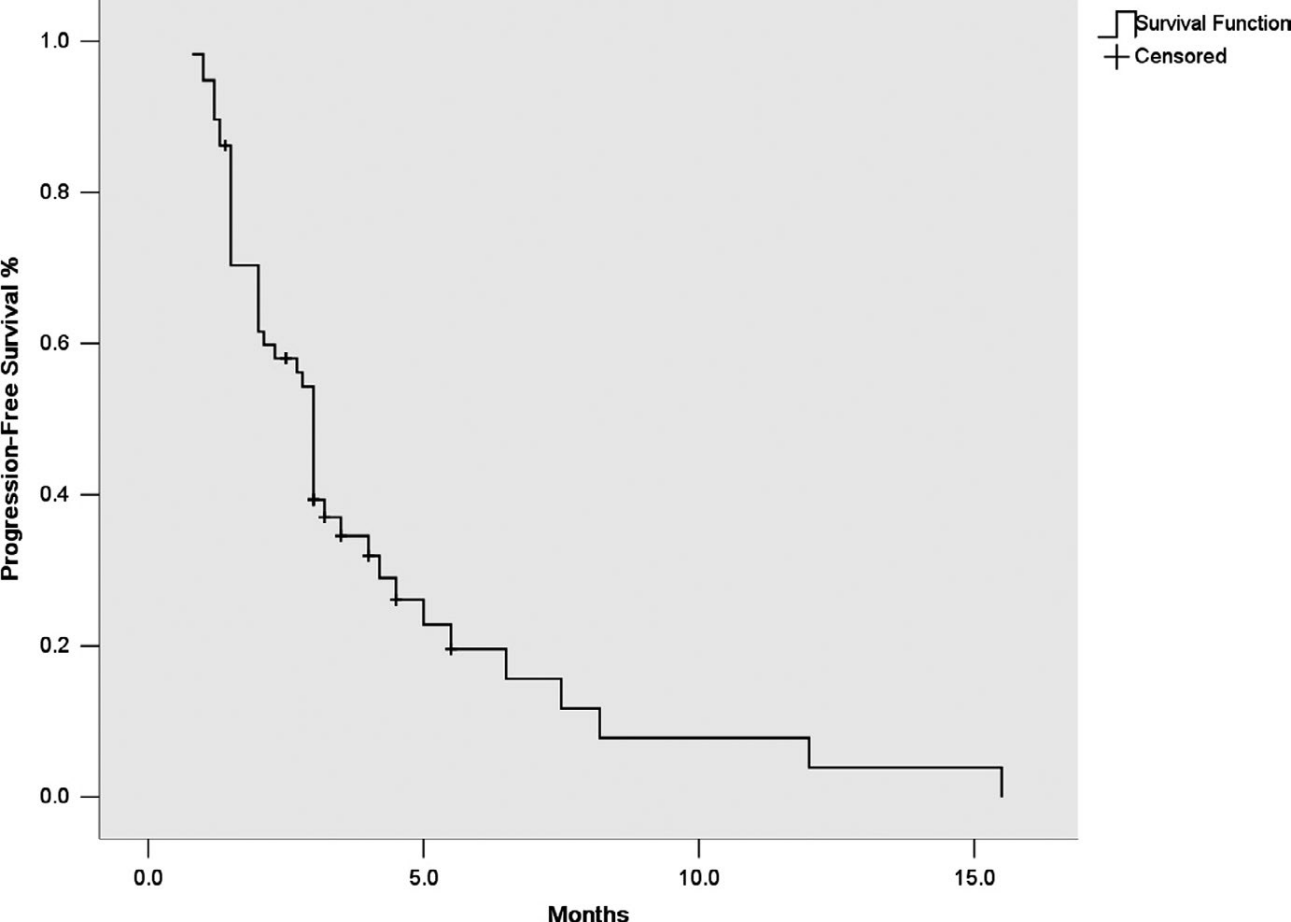
The Kaplan‐Meier curve of progression‐free survival of gastrointestinal stromal tumors patients receiving dasatinib as third line treatment

**FIGURE 2:**
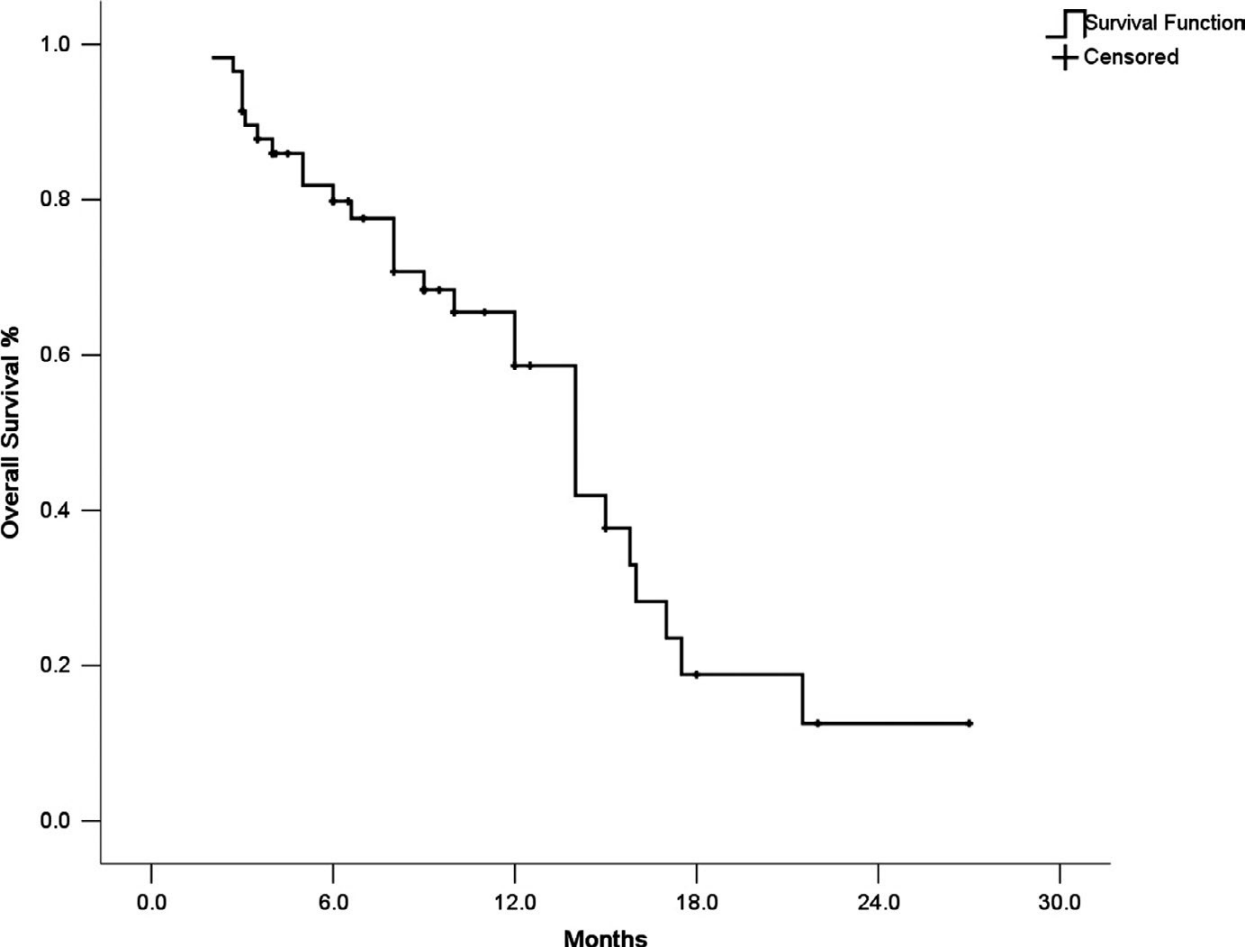
The Kaplan‐Meier curve of overall survival of gastrointestinal stromal tumors patients receiving dasatinib as third line treatment

The median duration of follow‐up was 22.0 months (95% CI: 14.5‐28.6 months). In all, 52 patients had GIST progression, 26 patients died, and 1 patient was lost to follow up. Among the 58 patients, 2 patients (3.4%) had partial response and 34 patients (58.6%) had stable disease. The 3‐month DCR was 36.2%. The median PFS was 3.1 months (95% CI, 2.77‐3.23 months) (Figure 1) and the median OS was 14.0 months (95% CI, 11.89‐16.1 months) (Figure 2). The 3‐month PFS rate was 53.4% and the 6‐month PFS rate was 13.8%.

### 3.3 Efficacy measures according to patient genotypes

In two patients who achieved PR, one had a gastric GIST with *KIT* exon 11 V560D mutation and secondary exon 17 N820Y mutation, and the other had jejunum GIST with primary *KIT* exon 17 N822K mutation. In four patients with *PDGFRA* D842V mutation, two patients had SD. A patient with secondary *KIT* exon 17 N822K mutation had a PFS of 15.5 months, and was still alive with OS reaching 27 months.

According to primary genotypes, the median PFS in GIST with *KIT* exon 11 mutation, exon 9 mutation, *PDGFRA* D842V mutation and wild type were 2.8 months (95% CI, 2.18‐3.42 months), 4.2 months (95% CI, 0.84‐7.56 months), 3.0 months (95% CI, 0.31‐5.70 months), and 5.0 months (95% CI, 3.309‐7.691 months), respectively. There was no statistically significant difference in PFS among patients of different genotypes (*P* = .851).

According to secondary genotypes, the median PFS in GIST with *KIT* secondary exon 13 or 14 mutation, secondary exon 17 mutation, no secondary mutation found and secondary mutation unknown were 3.0 months (95% CI, 1.243‐4.757 months), 3.0 months (95% CI, 2.759‐3.241 months), 3.0 months (95% CI, 1.623‐4.377 months), and 2.1 months (95% CI, 0.639‐3.561 months), respectively. No statistically significant difference was observed in PFS among patients of different secondary genotypes (*P* = .985).

### 3.4 Safety

The dose of dasatinib was not escalated to 70 mg twice daily in 3 (5.2%) patients due to grade 2 fatigue. In addition, four (6.9%) patients discontinued dasatinib therapy before the first efficacy evaluation due to AEs. In all, 18 (31.0%) patients temporarily discontinued treatment and 3 (5.2%) patients required dose reduction to 50 mg twice daily due to treatment‐emergent AEs (TEAEs). The most common AEs were anemia, proteinuria, fatigue, neutropenia, and diarrhea. Main grade 3 AEs included anemia (10.3%) and diarrhea (1.7%). It is worth noting that 10 patients (17.2%) happened grade 1 gastrointestinal bleeding during dasatinib treatment. Eight patients developed ascites or original ascites increased, but all of them accompanied with tumor progression. No treatment‐emergent death occurred in this study.

### 3.5 NGS in peripheral blood

#### 3.5.1 Concordance between liquid biopsy and tissue sequencing

NGS was undertaken of GIST tissues and blood samples in 21 patients and yielded identical results from the tissue and blood samples in six cases. Additional *KIT* mutations were detected in peripheral blood samples in six other cases including two cases with wild‐type GIST. However, no *KIT* or *PDGFRA* mutations were detected in peripheral blood in eight cases with gene mutation in tissue examination including exon 11 point mutation, exon 11 deletion, exon 9 duplication, and D842V point mutation (Table 2). Five of the eight cases had a low tumor burden.

**TABLE 2:** Consistency between liquid biopsy and tissue sequencing

Case	Primary mt (T)	Secondary mt (T)	Gene mt before dasatinib (B)
Case 1	Exon 11 557‐558 del	Exon 11 557‐558 del, exon 17 D820Y	Exon 17 D820Y
Case 2	Exon 11 V560D	Exon11 V560D, exon 17 N822K	Not detected
Case 3	Exon 11 V559D	Exon 11 V559D, exon 17 N822K	Not detected
Case 4	Exon 11 559‐565 del	Exon 11 559‐565 del, exon 17 R815I, exon 17 D816V	Exon 11 559‐565 del, exon 17 R815I, exon 17 D816V
Case 5	Exon 9 502‐503 dup	Exon 9 502‐503 dup	Exon 9 502‐503 dup
Case 6	Exon 11 557‐558 del	Not detected	Exon 11 557‐558 del, exon 17 N822K, exon 17 I817M
Case 7	Exon 11 Y568C	Exon 11 Y568C, exon 17 N822K	Exon 11 Y568C, exon 17 N822K, Exon 11 570‐576 del
Case 8	Exon 11 552‐570 del	Exon 17 N820Y	Not detected
Case 9	Exon 11 556‐559 del	Exon 11 556‐559 del, exon 13 V654A	Exon 11 556‐559 del, exon 13 V654A
Case 10	PDGFRA Exon 18 D842V	PDGFRA Exon 18 D842V	Not detected
Case 11	WT	WT	Exon 11 V559D
Case 12	Exon 11 V559D	Exon 11 V559D	Exon 11 V559D, exon 13 V654A
Case 13	Exon 11 V559D	Exon 11 V559D, exon 17 N822K	Exon 11 V559D, exon 17 N822K
Case 14	Exon 13 K642E	Exon 13 K642E	Exon 13 K642E, exon 13 V654A, exon 14 T670I
Case 15	Exon 9 502‐503 dup	Exon 9 502‐503 dup	Not detected
Case 16	Exon 11 W557S	Exon 11 W557S, exon 17 Y823D	Exon 11 W557S, exon 17 Y823D
Case 17	Exon 11 557‐561 del	Exon 11 557‐561 del, exon 17 Y823D	Exon 11 557‐561 del, exon 17 Y823D
Case 18	WT	WT	Exon 9 502‐503 dup
Case 19	Exon 9 502‐503 dup	Exon 9 502‐503 dup	Not detected
Case 20	Exon 17 N822K	Exon 17 N822K	Not detected
Case 21	Exon 11 557‐558 del	Exon 11 557‐558 del	Not detected

#### 3.5.2 SNV and CNV in 21 blood samples of GIST patients

**FIGURE 3:**
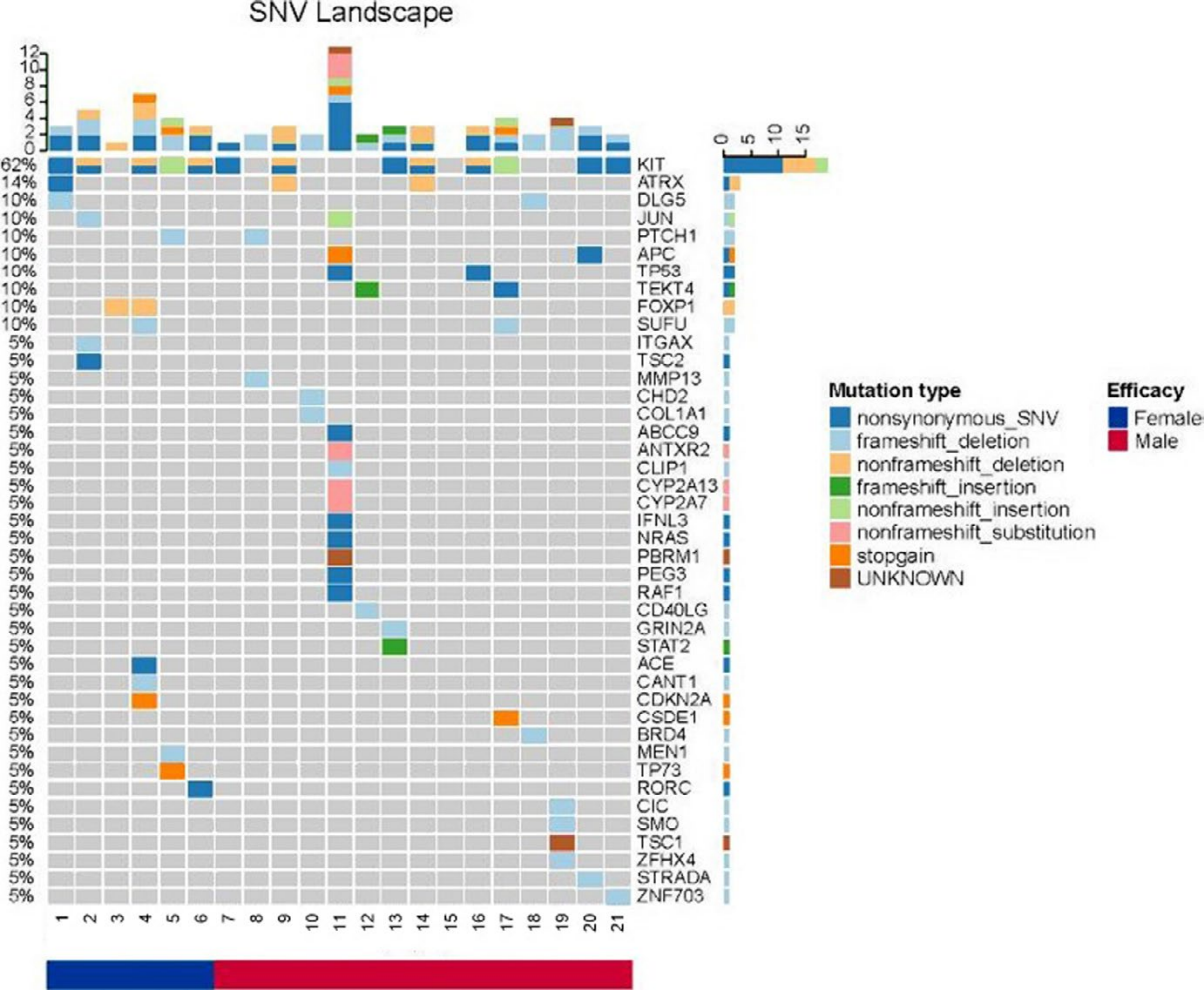
Single nucleotide variation (SNV) changes in the blood samples of 21 gastrointestinal stromal tumors patients

Among the 21 samples, all had at least one nonsynonymous or deletion variant in the captured genes. For 42 captured genes, *KIT* (61.9%), *ATRX* (14.3%), *DLG5* (9.5%), *JUN* (9.5%), *PTCH1* (9.5%), *APC* (9.5%), *TP53* (9.5%), *TEKT4* (9.5%), *FOXP1* (9.5%), and *SUFU* (9.5%) were the 10 most frequently SNV/Indel. Moreover, other variants were identified including *SRC* (4.8%), *NRAS* (4.8%), *CDKN2A* (4.8%), and *MEN1* (4.8%). In these common variations, only *ATRK* mutations were detected simultaneously with *KIT* mutations (Figure 3). There was no correlation between other variations and *KIT* mutations.

In 21 samples, 82 CNVs were detected. *SKT11* (76.2%), *SDHC* (52.4%), *CDKN2C* (42.9%), *TMEM127* (42.9%), *MAP2K3* (38.1%), *RECQL4* (28.6%), *PDPR* (19.0%), *NOTCH1* (14.3%), *FOXA1* (14.3%), and *ATM* (14.3%) were 10 ten most frequently CNVs. Other common CNVs were identified including *IL7R* (9.5%), *DDR2* (9.5%), *FCRL1* (9.5%), *SMARB1* (9.5%), *MLH1* (9.5%), *PTCH1* (9.5%), *NPM1* (9.5%), *SLC19A1* (9.5%), *RAD51B* (9.5%), and *FAT2* (9.5%). No statistical difference was observed in SNV, CNV, gender, or age.

Of the 21 patients, no secondary *Kit* mutation was detected either in the GIST tissue or peripheral blood samples of 8 patients. *SMAD3/4*, *FLT1*, *TSC1*, *STK11*, *MAP2K3*, *SMO*, *PTCH1*, *MLH1*, and *CDKN2C* variations were detected in the peripheral blood samples. These variations are mainly enriched in the RAS pathway and the PI3K pathway.

#### 3.5.3 The relationship of NGS results and efficacy of dasatinib

There was no statistical difference in SNV or CNV including *Kit* mutation between patients with PFS < 3 and ≥3 months. However, *APC* and/or *TP53* mutation was only detected in patients with PFS ≥ 3 months. On the contrary, *SUFU* and *TEKT4* mutation were found exclusively in patients with PFS < 3 months. The only one patient with *SRC* mutation had a PFS of 4.5 months.

Moreover, patients with OS < 12 months had a significantly higher proportion of *TP53* mutations than patients with OS > 12 months than (*P* = .012). In addition, patients with *SDHC* and *TMEM127* copy number loss had longer OS (*P* = .035, .006) compared with patients with normal copy number.

## 4 DISCUSSION

In phase I study and II clinical studies, dasatinib showed good antitumor activity, but had no obvious advantage over imatinib and sunitinib.^5^, ^6^ The inhibition by dasatinib of GISTs harboring *KIT* exon 17 mutation and *SRC* has led to more expectations for the drug as third‐line treatment. In this study, the 3‐month PFS rate was 53.4% in GIST patients receiving dasatinib therapy. However, in the study, patients received image examination every 6 weeks and some patients underwent imaging evaluation later than the planned 12 weeks after the start of dasatinib therapy. Therefore, the 3‐month PFS rate could be lower than 53.4%. In order to evaluate the PFS of dasatinib therapy more accurately, we analyzed the 6‐month PFS rate. Compared with the current standard third‐line treatment with regorafenib, dasatinib achieved a similar DCR, but both the 3‐ and 6‐month PFS rates and even OS were lower.^3^ Dasatinib showed moderate antitumor activity but failed to show the potential to surpass regorafenib as third‐line treatment of GIST. In safety analysis, the main TEAEs were anemia, proteinuria, fatigue, and diarrhea. Dose reductions and treatment interruptions occurred in a few patients, but TEAEs were tolerable. However, we need to be vigilant that a small number of patients happened gastrointestinal bleeding.

Dasatinib is a small molecule, adenosine triphosphate competitive inhibitor of *KIT*, *PDGFR*, and *SRC* family of kinases. Therefore, it is very important to evaluate the correlation between genotyping, especially secondary gene mutations, and the efficacy of dasatinib. In vitro studies have shown that dasatinib was less active against KIT activation loop mutant isoforms than against juxtamembrane domain mutant KIT except for D816Y mutation.^9^ Activated loop mutation is just the main type of *KIT* secondary mutation. However, unfortunately, neither primary nor secondary gene mutations were found to predict the efficacy of dasatinib in this study. The results of subgroup analysis in this study are consistent with those in vitro studies. This suggests that compared with the selective inhibition by sunitinib of tumors with secondary exon 13 V654A mutation^10^ and by regorafenib of tumors with secondary exon 17 mutation,^11^ dasatinib as third‐line treatment has no advantageous selectivity for *KIT* secondary mutation types. However, it was found that wild‐type GIST patients seemed to have longer PFS, showing that dasatinib as third‐line treatment may be more effective for this subgroup of patients. In addition, GIST with *PDGFRA* D842V mutation is considered to be resistant to almost all existing TKIs.^12^ In previous study, avapritinib has shown excellent efficacy for GISTs harboring D842V mutation.^13^ In a second‐line study, dasatinib was used to treat GIST patients with D842V mutation and PR was obtained in two cases.^6^ In the current study, 50% of D842V mutated GIST patients had SD after dasatinib therapy. Dasatinib could still be used as an alternative drug for the treatment of GIST patients with D842V mutation before avapritinib is approved.

In addition, effective treatment of wild‐type GIST suggested that inhibition of *SRC* or downstream signaling pathways of *KIT* could play another important role in the treatment of drug‐resistant GIST with dasatinib. In the second‐line treatment study of dasatinib, SRC‐positive patients showed better efficacy.^6^ In this study, no immunohistochemical staining of *SRC* was performed, but *SRC* mutation was found in peripheral blood sample of a patient by NGS. The PFS of this patient reached 4.5 months, which is better than the overall level.

In this study, 21 patients underwent NGS of peripheral blood samples to explore the role of activation of other pathways in resistance to GIST besides secondary *KIT* gene mutation and whether other gene mutations could predict the efficacy of dasatinib treatment. In the 21 cases, *KIT* gene mutation still accounted for the main proportion of mutations detected in the blood by NGS. At the same time, we detected some other SNVs such as *ATRX*, *JUN*, *PTCH1*, *TP53*, *TEKT4*, *FOXP1*, and *SUFU* and CNVs such as *STK11*, *SDHC*, and *CDKN2C* related to tumor signaling pathways. In the peripheral blood of eight patients without secondary gene mutation, we found that SNV and CNV mutations were mainly concentrated in the RAS pathway and the PI3K pathway. In previous studies, *RAS* mutation may be one of the pathogenesis of wild type GIST,^14^, ^15^ and activation of the PI3K pathway in drug‐resistant GIST was thought to be associated with secondary drug resistance.^16^, ^17^ Therefore, we speculate that the activations of these signaling pathways in blood may be other mechanisms of drug resistance besides *KIT* secondary mutations. In in vitro studies, dasatinib showed moderate effect on the PI3K/AKT pathway by inhibition of *SRC*.^18^ In this study, the 3‐month PFS of patients treated with dasatinib who had no gene mutation suggests that dasatinib may effective in the inhibition of KIT downstream signaling pathways.

We also analyzed the predictive power of peripheral blood gene variations on the efficacy of dasatinib. An interesting finding is that *TP53* mutations occur only in patients with longer PFS, while patients with *TP53* mutations had longer OS, which is inconsistent with previous results that *TP53* was considered a negative prognostic factor.^19^, ^20^ It was found that dasatinib combined with doxorubicin played a stronger role in chondrosarcoma with *TP53* mutation.^21^ In addition, GIST patients with *SDHC* and *TMEM127* copy number loss had longer OS. However, the natural history of *SDH* deficient tumors is rather indolent, with longer disease control time than other tumors with different mutations. Meanwhile, the results were from a small sample size. Therefore, we cannot draw the conclusion that *SDHC* and *TMEM127* copy number loss are related to the efficacy of dasatinib treatment.

Compared with NSG using tumor tissues, one of the main advantages of NSG using liquid biopsy is that it can overcome the heterogeneity of tumors and detect other mutant genes which are not found in a single tissue sample. This is also confirmed in this study. In 21 patients, the concordance of *KIT/PDGFRA* between tissue and peripheral blood samples was 61.9%. In 28.6% of the patients, new *KIT* mutations were detected in the peripheral blood, including secondary gene mutations that were not detected by tissue examination, which is very helpful to guide drug therapy. However, *KIT/PDGFRA* mutations were not detected in peripheral blood of eight patients, and five of them had a low tumor load. The failure to detect the mutations may be related to the insufficient content of GIST circulating tumor DNA in the peripheral blood. In previous studies, it has been proved that it is difficult to detect circulating tumor DNA in GIST with too low a tumor load.^22^ In the GRID trial, the concordance rate was 84% between plasma and tissue for detection of primary *KIT* mutations and Beads Emulsion Amplification Magnetics technology was less sensitive for the detection of primary *KIT* exon 11 mutations in plasma DNA.^23^ Therefore, at this stage, liquid biopsy could not replace tumor tissue sequencing for GIS T genotype analysis.

In summary, dasatinib shows moderate activity in metastatic GIST, and the toxicities are tolerable. Dasatinib could offer a therapeutic option for patients who cannot take regorafenib, especially those with wild‐type GIST or D842V mutation GIST. Our NGS data suggest that dasatinib may also suppress signaling pathways apart from *KIT*, which warrants further study.

## CONFLICT OF INTEREST

All the authors report no conflicts of interest in this work.

## AUTHORS' CONTRIBUTION

Jian Li and Lin Shen are responsible for designation, quality control, data analysis, and article revision. Ye Zhou contribute to subject screening, quality control, and article writing. Xinhua Zhang, Xiaojun Wu, Yongjian Zhou, Bo Zhang, Xiufeng Liu, and Xin Wu are responsible for subject screening and quality control as PI of different centers. Yan Li is responsible for tumor sample collection. Ye Zhou, Xinhua Zhang, Xiaojun Wu, Yongjian Zhou, Bo Zhang, Xiufeng Liu, and Xin Wu contribute equally to this work.

## Data Availability

The datasets generated and/or analyzed during the current study are not publicly available to ensure patient privacy, but are available from the corresponding author on reasonable request.

## References

[1] Demetri GD , von Mehren M , Blanke CD , et al. Efficacy and safety of imatinib mesylate in advanced gastrointestinal stromal tumors. N Engl J Med. 2002;347(7):472‐480. 12181401 10.1056/NEJMoa020461

[2] Demetri GD , van Oosterom AT , Garrett CR , et al. Efficacy and safety of sunitinib inpatients with advanced gastrointestinal stromal tumour afterfailure of imatinib: a randomised controlled trial. Lancet. 2006;368(9544):1329‐1338. 17046465 10.1016/S0140-6736(06)69446-4

[3] Demetri GD , Reichardt P , Kang YK , et al. Efficacy and safety of regorafenib foradvanced gastrointestinal stromal tumours after failure of imatinib and sunitinib (GRID): an international, multicentre, randomised, placebo‐controlled, phase 3 trial. Lancet. 2013;381(9863):295‐302. 23177515 10.1016/S0140-6736(12)61857-1 PMC3819942

[4] Gnoni A , Marech I , Silvestris N , Vacca A , Lorusso V . Dasatinib: an anti‐tumour agent via srcinhibition. Curr Drug Targets. 2011;12(4):563‐578. 21226671 10.2174/138945011794751591

[5] Montemurro M , Cioffi A , Dômont J , et al. Long‐term outcome of dasatinib first‐line treatment in gastrointestinal stromal tumor: a multicenter, 2‐stage phase 2 trial (Swiss Group for Clinical Cancer Research 56/07). Cancer. 2018;124(7):1449‐1454. 29315500 10.1002/cncr.31234

[6] Schuetze SM , Bolejack V , Thomas DG , et al. Association of dasatinib with progression free survival among patients with advanced gastrointestinal stromal tumors resistant to imatinib. JAMA Oncol. 2018;4(6):814‐820. 29710216 10.1001/jamaoncol.2018.0601 PMC6145709

[7] Chan AW , Tetzlaff JM , Altman DG , et al. SPIRIT 2013 statement: defining standard protocol items for clinical trials. Intern Med. 2013;158(3):200‐207. 10.7326/0003-4819-158-3-201302050-00583 PMC5114123 23295957

[8] Schulz KF , Altman DG , Moher D , CONSORT Group . CONSORT 2010 statement: updated guidelines for reporting parallel group randomised trials. BMJ. 2010;340:c332. 20332509 10.1136/bmj.c332 PMC2844940

[9] Hsueh YS , Lin CL , Chiang NJ , et al. Selecting tyrosine kinase inhibitors for gastrointestinal stromal tumor with secondary KIT activation‐loop domain mutations. PLoS ONE. 2013;8(6):e65762. 23840364 10.1371/journal.pone.0065762 PMC3688691

[10] Heinrich MC , Maki RG , Corless CL , et al. Primary and secondary kinase genotypes correlate with the biological and clinical activity of sunitinib in imatinib‐resistant gastrointestinal stromal tumor. J Clin Oncol. 2008;26(33):5352‐5359. 18955458 10.1200/JCO.2007.15.7461 PMC2651076

[11] Yeh CN , Chen MH , Chen YY , et al. A phase II trial of regorafenib in patients with metastatic and/or a unresectable gastrointestinalstromal tumor harboring secondary mutations of exon 17. Oncotarget. 2017;8(27):44121‐44130. 28487491 10.18632/oncotarget.17310 PMC5546467

[12] Corless CL , Schroeder A , Griffith D , et al. PDGFRA mutation in gastrointestinal stromal tumors: frequency, spectrum and in vitro sensitivity to imatinib. J Clin Oncol. 2005;23(23):5357‐5364. 15928335 10.1200/JCO.2005.14.068

[13] Evans EK , Gardino AK , Kim JL , et al. A precision therapy against cancers driven by KIT/PDGFRA mutations. Sci Transl Med. 2017;9(414). pii: eaao 1690–1701. 10.1126/scitranslmed.aao1690 29093181

[14] Nannini M , Astolfi A , Urbini M , et al. Integrated genomic study of quadruple‐WT GIST (KIT/PDGFRA/SDH/RAS pathway wild‐type GIST). BMC Cancer. 2014;20(14):685. 10.1186/1471-2407-14-685 PMC4181714 25239601

[15] Miranda C , Nucifora M , Molinari F , et al. KRAS and BRAF mutations predict primary resistance to imatinib in gastrointestinal stromal tumors. Clin Cancer Res. 2012;18(6):1769‐1776. 22282465 10.1158/1078-0432.CCR-11-2230

[16] Bosbach B , Rossi F , Yozgat Y , et al. Direct engagement of the PI3K pathway by mutant KIT dominates oncogenic signaling in gastrointestinal stromal tumor. Proc Natl Acad Sci USA. 2017;114(40):E8448‐E8457. 28923937 10.1073/pnas.1711449114 PMC5635919

[17] Long ZW , Wu JH , Cai‐Hong Wang YN , Zhou Y . iR‐374b promotes proliferation and inhibits apoptosis of human GIST cells by inhibiting PTEN through activation of the PI3K/Akt pathway. Mol Cells. 2018;41(6):532‐544. 29902839 10.14348/molcells.2018.2211 PMC6030239

[18] Beadnell TC , Nassar KW , Rose MM , et al. Src‐mediated regulation of the PI3K pathway in advanced papillary and anaplastic thyroid cancer. Oncogenesis. 2018;7(2):23. 29487290 10.1038/s41389-017-0015-5 PMC5833015

[19] Offin M , Chan JM , Tenet M , et al. Concurrent RB1 and TP53 alterations define a subset of EGFR‐mutant lung cancers at risk for histologic transformation and inferior clinical outcomes. J Thorac Oncol. 2019;14(10):1784–1793. 31228622 10.1016/j.jtho.2019.06.002 PMC6764905

[20] Kawaguchi Y , Kopetz S , Newhook TE , et al. Mutation status of RAS, TP53, and SMAD4 is superior to mutation status of RAS alone for predicting prognosis after resection of colorectal liver metastases. Clin Cancer Res. 2019;25(19):5843–5851. 31221662 10.1158/1078-0432.CCR-19-0863 PMC6774854

[21] van Oosterwijk JG , van Ruler MA , Briaire‐de Bruijn IH , et al. Src kinases in chondrosarcoma chemoresistance and migration: dasatinib sensitises to doxorubicin in TP53 mutant cells. Br J Cancer. 2013;109(5):1214‐1222. 23922104 10.1038/bjc.2013.451 PMC3778302

[22] Xu H , Chen L , Shao Y , et al. Clinical application of circulating tumor DNA in the genetic analysis of patients with advanced GIST. Mol Cancer Ther. 2018;17(1):290‐296. 29133619 10.1158/1535-7163.MCT-17-0436

[23] Reichardt P , Demetri G , Kang Y‐K , et al. Mutational analysis of plasma DNA from patients (pts) in the phase III GRID study of regorafenib (REG) versus placebo (PL) in tyrosine kinase inhibitor (TKI)‐refractory GIST: correlating genotype with clinical outcomes. Onkologie. 2013;36(7):179‐180.

